# Feedback from recently returned veterans on an anonymous web-based brief alcohol intervention

**DOI:** 10.1186/1940-0640-7-17

**Published:** 2012-08-28

**Authors:** Gwen T Lapham, Eric J Hawkins, Laura J Chavez, Carol E Achtmeyer, Emily C Williams, Rachel M Thomas, Evette J Ludman, Kypros Kypri, Stephen C Hunt, Katharine A Bradley

**Affiliations:** 1 Health Services Research & Development (HSR&D), Northwest Center of Excellence, Veterans Affairs (VA) Puget Sound Health Care System, 1100 Olive Way, Suite 1400, Seattle, WA, 98101, USA; 2 Center of Excellence in Substance Abuse Treatment and Education (CESATE), 1660 South Columbian Way, Seattle, WA, 98108, USA; 3 General Medicine Service, Veterans Affairs (VA) Puget Sound Health Care System, 1660 South Columbian Way, Seattle, WA, 98108, USA; 4 Department of Medicine, University of Washington, Box 356420, Seattle, WA, 98195, USA; 5 Department of Health Services, University of Washington, Box 357660, Seattle, WA, 98195, USA; 6 Group Health Research Institute, 1730 Minor Ave., Suite 1600, Seattle, WA, 98101, USA; 7 School of Medicine and Public Health, University of Newcastle, Level 3, David Maddison Clinical Sciences Building, Callaghan, New South Whales, 2308, Australia

**Keywords:** Internet, Alcohol, Brief intervention, Feedback, Iraq war, Veteran

## Abstract

**Background:**

Veterans of Operation Enduring Freedom and Operation Iraqi Freedom (OEF/OIF) are at increased risk for alcohol misuse, and innovative methods are needed to improve their access to alcohol screening and brief interventions (SBI). This study adapted an electronic SBI (e-SBI) website shown to be efficacious in college students for OEF/OIF veterans and reported findings from interviews with OEF/OIF veterans about their impressions of the e-SBI.

**Methods:**

Outpatient veterans of OEF/OIF who drank ≥3 days in the past week were recruited from a US Department of Veterans Affairs (VA) Deployment Health Clinic waiting room. Veterans privately pretested the anonymous e-SBI then completed individual semistructured audio-recorded interviews. Their responses were analyzed using template analysis to explore domains identified *a priori* as well as emergent domains.

**Results:**

During interviews, all nine OEF/OIF veterans (1 woman and 8 men) indicated they had received feedback for risky alcohol consumption. Participants generally liked the standard-drinks image, alcohol-related caloric and monetary feedback, and the website’s brevity and anonymity (*a priori* domains). They also experienced challenges with portions of the e-SBI assessment and viewed feedback regarding alcohol risk and normative drinking as problematic, but described potential benefits derived from the e-SBI (emergent domains). The most appealing e-SBIs would ensure anonymity and provide personalized transparent feedback about alcohol-related risk, consideration of the context for drinking, strategies to reduce drinking, and additional resources for veterans with more severe alcohol misuse.

**Conclusions:**

Results of this qualitative exploratory study suggest e-SBI may be an acceptable strategy for increasing OEF/OIF veteran access to evidenced-based alcohol SBI.

## Introduction

Individuals deployed for combat in Iraq and Afghanistan for Operation Enduring Freedom and Operation Iraqi Freedom (OEF/OIF), a group now numbering more than 2.2 million [1], are at high risk for alcohol misuse [2], and those with combat exposure are especially at risk for new onset of alcohol misuse and alcohol-related problems [2-4]. The prevalence of alcohol misuse among OEF/OIF veterans (22–40%) is highest among veterans treated in the Veterans Health Administration (VA) [5-7]. Yet, OEF/OIF veterans are cautious about seeking care for substance use and mental-health concerns [2,8,9].

Evidence-based alcohol screening and brief alcohol intervention (SBI) can reduce drinking [10]. The VA implemented routine clinical alcohol screening [11] and brief intervention (BI) after implementation of a performance measure and electronic decision support [12]. However, false-negative screens [13], receipt of care outside the VA, and stigma-related concerns [8,14] prevent many OEF/OIF veterans from accessing alcohol-related care. Innovative approaches are needed to increase the reach of SBI for OEF/OIF veterans.

Web-based alcohol screening and BI (e-SBI) can increase access to evidenced-based care for alcohol misuse by providing anonymous personalized interventions to large numbers of people at low cost [15-17] and can increase disclosure of alcohol use[18]. An anonymous web-based e-SBI program may be particularly well-suited for young, employed, and web-savvy OEF/OIF veterans [7,19] who have a preference for online mental-health information and may be more comfortable with a private e-SBI due to the stigmatization of alcohol misuse [19-21].

Electronically delivered SBIs vary widely in length, therapeutic intensity, design, and populations targeted, and systematic reviews have arrived at different conclusions about their overall efficacy [22-26]. A recent meta-analysis of 19 randomized control trials found e-SBI resulted in a similar reduction in weekly alcohol consumption as face-to-face brief intervention (BI) [27]. A brief (<10 minute) single-session e-SBI known as THRIVE has proven efficacious in New Zealand and Australian college students [28,29] and includes alcohol-use assessment and feedback based on the 10-item Alcohol Use Disorders Identification Test (AUDIT) [30]. THRIVE’s potential causal mechanism has been described as motivational enhancement through the provision of self-focused and normative feedback [31,32].

Because of THRIVE’s proven efficacy in young adults, the current study adapted its web interface and programming logic [29,33] for use among OEF/OIF veterans based on feedback from VA and non-VA alcohol experts and local and national OEF/OIF veteran experts who were interviewed after reviewing the THRIVE website (Table 1). The resulting e-SBI (http://www.DrinkCheck.org) used a briefer alcohol screen, the 3-item AUDIT-C (consumption questions only) questionnaire [11,34] because of the availability of normative AUDIT-C data from VA outpatients. DrinkCheck was equally as brief (<10 minutes) and included 12 web pages (Table 1). This qualitative study used semistructured interviews to explore a small sample of OEF/OIF veteran patients’ experiences with, and opinions about, DrinkCheck. 

**Table 1:** Features of THRIVE and corresponding adaptations for DrinkCheck

**Features/Content**	**THRIVE**	**DrinkCheck**
**DESIGN**		
**Length/design**	Brief single-session; 1 intro, 7 assessment, 1 feedback, and 3 optional resources web pages	Brief single-session; 1 intro, 5 assessment, 5 feedback, and 1 optional resource web-page; civilian & military photos
**Standard drinks image**	Number of drinks in an alcoholic beverage by type and packaging	Equivalent of 1 standard US drink by type
**ASSESSMENT**		
**Patient characteristics**	Age, gender, weight, height	Age, gender, weight
**Drinking in past year**	10-item AUDIT	3-item AUDIT-C plus question regarding typical choice of alcoholic beverages
**Drinking in last 4 weeks**	Largest number of drinks consumed on one occasion and duration of episode	Unchanged
**Additional alcohol-related questions**	Questions regarding other students drinking and alcohol labeling	Replaced with frequency of alcohol-related health and relationship concerns in past four weeks
**Tobacco use**	Assessment of current use	Removed for simplification and brevity
**PERSONALIZED FEEDBACK**		
**Alcohol-risk category**	‘Non-hazardous,’ ‘hazardous,’ ‘harmful,’ and ‘dependent’ drinking based on AUDIT score	‘Low-risk,’ ‘risky,’ ‘high-risk’ and ‘very high-risk’ drinking based on AUDIT-C score and reported consumption above NIAAA recommended limits^*^
**Normative feedback (age- & gender-based)**	Above recommended limits drinking compared to Australian college students (typical drinks/occasion and typical drinks/week)	Participant heavy episodic drinking^**^ compared to AUDIT-C responses from age- and gender-matched VA outpatients† (maximum drinks per one occasion and % of patients drinking less)
**Health and relationship concerns**	Not part of feedback	Primary concerns in past 4 weeks reported as part of brief alcohol-related health feedback
**Alcohol calories**	Not part of feedback	Weekly calories along with the equivalent in cheeseburgers and hours of exercise
**Blood alcohol concentration**	Reported for highest drinking occasion in past 4 weeks along with likelihood of vehicular death	Reported for highest drinking occasion in past 4 weeks along with legal driving limit; adapted to US measures
**Money spent on alcohol**	Estimated for past year	Estimated for past year; adapted to US measures
**Resources (non-personalized)**	Three optional pages for alcohol-related ‘Facts’, ‘Tips’ and ‘Support’	One page of VA and non-VA alcohol- and treatment-related resources, including links to NIAAA’s “Rethinking Drinking”

^*^National Institute on Alcohol Abuse and Alcoholism (NIAAA) recommended drinking limits: ≤14 drinks per week and <5drinks per occasion for men; ≤7 drinks per week & <4 drinks per occasion for women.

^**^Participant heavy episodic drinking (≥5 drinks per occasion for men, ≥4 drinks per occasion for women) was based on AUDIT-C responses and drinking in the last 4 weeks.

^†^From mailed VA outpatient surveys between 2004–2007.

## Methods

### Adapted e-SBI

**Figure 1:**
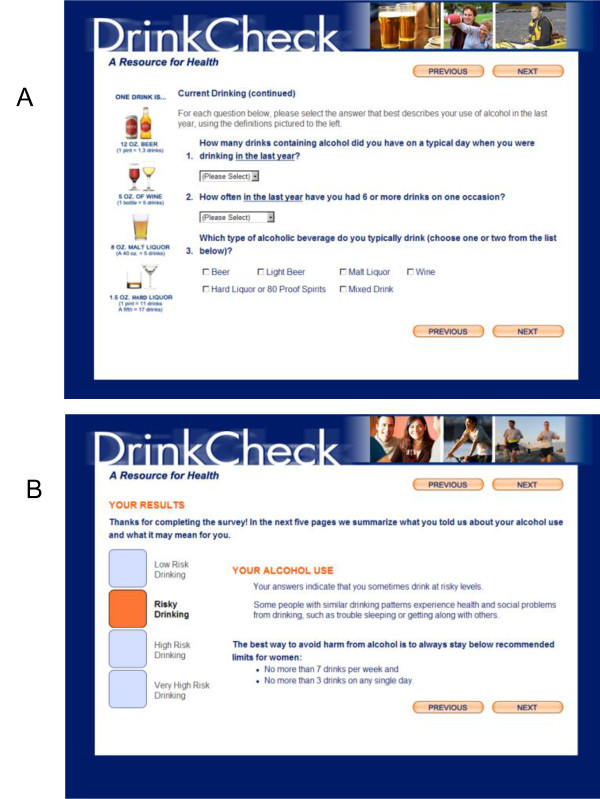
**Screenshots of the DrinkCheck e-SBI tool adapted for OEF/OIF veterans.A**—alcohol assessment questions; **B**—initial feedback page.

Changes made to THRIVE in developing DrinkCheck are outlined in Table 1. In brief, DrinkCheck assessed frequency and quantity of alcohol use (Figure 1A) and assigned participant alcohol risk to one of four categories based on AUDIT-C scores, estimated weekly alcohol consumption, and the greatest number of drinks per day or occasion. Participants were provided brief feedback on their level of risk (Figure 1B), and, if participants reported heavy episodic drinking (≥5 drinks per occasion for men and ≥4 drinks per occasion for women), they received normative feedback that compared their drinking to that of age- and gender-matched VA outpatients based on AUDIT-C data from outpatient surveys (2004–2007) [35]. Additional personalized feedback was offered as well (Table 1). 

### Participant selection

A convenience sample of patients attending the Deployment Health Clinic (DHC) of a single large urban VA medical center was approached in the waiting room and asked to participate in a study of a short anonymous web-based alcohol-use assessment and feedback program for returning veterans. Interested patients completed a brief eligibility survey and were eligible if they were OEF/OIF veterans and reported drinking alcohol on three or more days in the past week. Patients were excluded if they were pregnant or demonstrated major cognitive impairments on the Mini-Cog assessment instrument [36]. Eligible patients were offered US $20 in compensation for pretesting the DrinkCheck and completing interviews. The VA “rapid-response” research program that funded this study did not allow studies of 10 or more veterans; a larger sample would have required review by the US Office of Management and Budget and delayed results [37,38]. Therefore, enrollment was limited to nine subjects. Patients provided verbal consent, and the study received approval and waivers of written informed consent and Health Insurance Portability and Accountability Act (HIPAA) authorization from the VA Puget Sound Health Care System Institutional Review Board. To prevent participant identification due to the small sample size and ensure participant confidence in their privacy, minimal demographic information was collected.

### Data collection

Participants completed DrinkCheck alone in a private clinic room on a freestanding laptop that was not connected to the internet. Subsequently, participant responses were cleared so that the interviewer was blinded to responses unless volunteered. Participants were then interviewed by the lead author using a semistructured interview that asked about their experience completing the e-SBI program (Appendix A). Participants were asked to review and provide their opinions on each page of DrinkCheck, as well as their thoughts on how to make it more appealing to other returning veterans. Initial questions included, “How did you feel about the feedback you received?” and then for each page, “What are your impressions of this page?” followed by additional open-ended questions to explore responses and any concerns they raised. Interviews were digitally audio-recorded, transcribed, and reviewed for accuracy.

### Data analysis

Two of the coinvestigators, a clinical psychologist and a nonclinician researcher, used template analysis [39,40] to initially code the transcribed interviews. Template analysis is a qualitative technique that lies between grounded theory and content analysis and begins with an inventory of *a priori* domains expected to be strongly relevant. For this study, *a priori* domains reflected the anonymity and individual features of DrinkCheck [33,41]. Emergent domains were those that emerged from the data as coding proceeded (e.g., those not identified *a priori*) and were extensively and iteratively reviewed with investigators. A final coding template was arrived at by consensus including both *a priori* and emergent domains.

## Results

### Participants

Thirty-eight patients were approached in the waiting room of the DHC, and 36 (95%) agreed to participate. Of the 36, 17 (47%) were eligible OEF/OIF veterans who reported consuming alcohol on three or more days in the previous week. Eight of 17 (47%) eligible patients either did not have time to participate following their appointment or did not return from the clinic, which was on a different floor than the waiting room. One female and eight male OEF/OIF veterans representing multiple military service branches (Table 2) and a broad age range (23–55 years; mean, 33 years) completed the study. During the interview, each participant volunteered that their drinking was categorized as risky, high-risk, or very high-risk by the e-SBI feedback and that they had also received feedback on heavy episodic drinking. To avoid identification of the one female participant, participants are hereafter referred to as male.

**Table 2:** Operation Enduring Freedom (OEF)/Operation Iraqi Freedom (OIF) veteran participant characteristics

**Veteran**	**Military Branch**	**War Served**	**Days of Consumption (past week)**
A	Navy	OIF	3
B	Army	OIF	6
C	Army	OIF	3
D	Navy	OEF & OIF	3
E	Army National Guard	OEF & OIF	5
F	Marine Corps	OEF & OIF	3
G	Navy	OEF	5
H	Army	OIF	6
I	Army National Guard	OIF	3

### *A priori* domains

Participants provided useful feedback on six *a priori* domains regarding features of DrinkCheck (Table 3). Overall, they were pleased with its length, standard drinks image, and feedback on alcohol calories and money spent on alcohol. They were not surprised by the feedback on blood alcohol concentration and legal driving limits. They also reported the anonymity of DrinkCheck was important for obtaining truthful responses.

**Table 3:** ***A Priori*Domains and Selected Quotes from Interviews with Nine Operation Enduring Freedom (OEF)/Operation Iraqi Freedom (OIF) Veterans**

***A priori*domains**	**Quotes**
The e-SBI program was considered short and succinct	*“I mean, it’s good ‘cause it’s not too long, and it’s not too short… it has good information and is something, you know, a soldier or anybody can, could, actually take.”* (Veteran C)
	*“It was short, simple, to the point, which is always easiest with military people - very impatient, just get to the point.”* (Veteran G)
The standard drinks image was considered helpful	*“When I saw these pictures on the side with the equal amounts of drinks to a bottle or a pint, it was kind of interesting ‘cause I didn’t really know that… made me think of, actually, how much I really drink.”* (Veteran C)
	*“That is actually very informative ‘cause a lot of people don’t know exactly, I think, how much one drink is. Depending on what bar you go to or how many you drink at home, people can distinguish one drink as an entire glass of whiskey or… as a 24-ounce can of beer.”* (Veteran E)
Participants appreciated feedback on estimated alcohol calories consumed and hours of exercise	*“I was appalled at how much calories I had consumed…it was just like ‘Oh, geez.’”* (Veteran G)
	*“It was cool. I was like ‘Whoa, it shows, like, how much…food and stuff, like, it could be equaled out to’…I’d rather have a cheeseburger, obviously.”* (Veteran I)
Feedback on estimated money spent on alcohol in the past year was also appreciated	*“I said ‘Thank goodness I don’t go to the bar.’ But, wow…mine was, like, $700. I said ‘I could have bought a TV or something.’ You know…you could buy a used car… I liked that…I wouldn’t change that. That’s good.”* (Veteran D)
	*“That’s a lot of money…right now I’m unemployed so it’s like do I really need to be drinking that much?”* (Veteran G)
	*“It’s like ‘Dang. I don’t want to spend that much on that, that stuff.’ You know? Like, I could be spending that on other things that, you know, I won’t be getting a headache in the morning.”* (Veteran I)
Feedback on the highest blood alcohol content and legal driving limit was obvious, but some noted it was worth keeping	*“When I drank that much I was drunk, I remember. That’s pretty obvious too, I’d have to say. I didn’t plan on driving ‘cause I knew I was drunk…I think that was a good one.”* (Veteran F)
	*“The point is well taken. But, I knew that anyway. Not saying that it shouldn’t be in here. I’m just saying that ‘Well, yeah, I had 12 drinks in four hours. Of course I was trashed.’…And is it illegal to drive? Bet your butt it’s illegal to drive…I think it’s good to be in there.”* (Veteran H)
The anonymity of the eSBI website was noted as a benefit	*“I think if it was private and the information was solely provided to the individual…you might have a better impact on that individual…for them to be more open and truthful about themselves.”* (Veteran D)
	*“I think on NKO* [Navy Knowledge Online]*…if they had asked me specific things like that and it would have gone in electronic record, I would have definitely not answered honestly….so, knowing that it’s anonymous helps people answer honestly, and knowing that it’s not going to go basically bite them in the ass at some point”* (Veteran G)

### Emergent domains

Seven emergent domains were identified from participant interviews and are described below.

### Questions about alcohol consumption “in the past year” were difficult to answer for recently returned veterans

Veterans reported alcohol was scarce and was generally prohibited during deployment. As a result, most participants said they did not drink alcohol while serving overseas. However, the AUDIT-C questions assessed typical and heavy-episodic drinking in the past year and were therefore challenging for participants who had recently returned:

"[Veteran C]: *I guess the timing on it… it plays a major factor, because at some points in the year, you actually don’t even drink.*"

Participants were aware that their drinking when not deployed was probably most pertinent to the assessment, yet many averaged their consumption over the entire year:

"[Veteran E]: *But over the past year people’s situations will be different.... I was just on deployment, so for six months I didn’t have anything… and then this past six months, it’s like, “Well, OK, now I’m getting back into the swing of things.” And so, they average it out over that, that entire time.*"

### Questions about health and relationship concerns were confusing to veterans

DrinkCheck included questions about the frequency of health and relationship concerns in the past four weeks. Despite an introductory statement, “These questions ask about symptoms and concerns veterans may have that can be influenced by drinking,” veterans did not understand why they were being asked about such concerns:

"[Veteran H]: *So, the question is, okay, “How often are you bothered by the following?” Well, why do you care?… What is it you’re trying to get at when you ask me these questions?*"

Additionally, participants wondered how and in which causal direction the concerns were associated with alcohol:

"[Veteran B]: *Some of these I also look as being effect, not so much a cause.... “Managing pain”—don’t really see it so much as an effect as a cause. “Trouble falling asleep, staying asleep or nightmares” can actually go both sides.*"

"[Veteran F]: *Those were all pretty good questions that apply to drinking. I answered them honestly, but… some of those you can have whether you’re drinking or not.*"

### Veterans wanted transparent nonjudgmental feedback and practical advice

The alcohol-risk feedback posed several problems for participants. First, they did not understand how or why they were assigned to their particular risk category:

"[Veteran B]: *It doesn’t show me what it is that I do that put me in that risk category versus the next one down or the one below that.... What makes me very high risk? You know, outline it* [referring to risk categories]. *What about me makes me so much higher risk than the person in this one?*"

"[Veteran D]: *We didn’t go into depth about the category you’re putting me in….what do I do? Why does it matter?… You know, am I going to die that week because I had 15 drinks?*"

Overall, participants wanted an explanation of what specifically about their drinking was risky as well as risk-reduction strategies tailored to their alcohol consumption and problems. Veteran D suggested adding more detail to the risk categories (e.g., “probably needs no assistance,” “should seek assistance”), as he wanted to know what he could do to reduce his risk:

"*Okay, so, yeah, I drink too much. I’ve thrown up. I’ve had a hangover. That’s risky.... And, I’m aware of that. [It] doesn’t tell me maybe I should do something different. Doesn’t motivate me to.*"

Veteran F explained how greater personalization could encourage a decrease in drinking:

"[Referring to risk assignment]: *If you saw that and were thinking about what could make my relationship better, and it says ‘less drinking,’ you’d be like, “Oh, well, there’s an eye-opener.” …[I]f it said something personal that could really only apply to you, by taking this, then I think people would think twice about going and drinking a lot.*"

Further, for some veterans, the risk feedback implied a judgment about them and their drinking:

"[Veteran B]: *[People] use this and it just turns back and says, “Yeah, you’re drinking too much.” And, all of a sudden, what has that done? Just one more person accusing them.*"

Although the risk categories were intended to highlight the continuum of alcohol-related risk, some veterans viewed alcohol risk as “all or nothing”:

"[Veteran I]: *If society says that you drink too much, then you’re deemed an alcoholic or a drunk. And, you know, it’s like there’s no real safe gray area in there that you could play with all that much.*"

### Veterans felt the context for their drinking should be considered when assessing risk

Participants did not typically consider their alcohol consumption as risky or inappropriate for the occasion and thought the context for drinking and whether problems were present, not just the level of consumption, were relevant to the assessment of risk:

"[Veteran A]: *Maybe it‘s just me, but maybe the wording ‘risky drinking.’ You know, you’re doing risky drinking. Well, it doesn’t seem risky when I’m doing it. (Laughter) I’m not going to hurt anybody. I’m not going to do anything wrong.*"

The context for risky drinking was perceived as particularly relevant if drinking was associated with a festive occasion:

"[Veteran E]: *[It’s been] my birthday, then Christmas, then New Year’s, all in two weeks. And, I’m always responsible with my drinking. I always make sure that if I, I’ve had more than three drinks total in the night, I always take it easy at least an hour before I drive. Make sure I’m calmed down.*"

Context was also considered particularly relevant for a difficult occasion:

"[Veteran G]: *For me, once a month going out and doing 13 drinks over 9 hours. Like, that’s not much compared to the two or three drinks I’ll have once or twice a week… Usually when I do have those binge nights, there’s a very specific reason I go out and drink like that, whether I’m celebrating… but this last time it was—I didn’t get a job, and I was very upset, and I thought I was gonna get it and it’s been two months, and I’m just like, uh. So, there was a reason; it was, “I’m just gonna go out and do this, and get all my frustrations out.”*"

Individual differences were also important for explaining heavy episodic drinking:

"[Veteran F, referring to risk feedback]: *That didn’t scare me into drinking any less ‘cause, honestly, I think people’s recommended limits aren’t everybody’s. ‘Cause people are, like, “Oh, don’t drink any more than a six pack.” And, that’s pretty much what I like. And, I don’t really seem to be having a problem with it.*"

### Veterans dismissed the normative feedback because it lacked credibility

All participants voluntarily reported receiving normative feedback for heavy episodic drinking, which attempted to highlight the incongruence between participants’ perception of “normal” drinking and actual norms. However, they had difficulty accepting veteran outpatients as an appropriate comparison group given differences in experiences among veterans:

"[Veteran E]: *If you’re just taking into account all VA patients 30 to 39 over the entire country, doesn’t necessarily mean that they spent as much time on deployment, doesn’t mean that they went to the same places that we did, or that I have.*"

Moreover, a few were skeptical of the comparison group data, believing it underrepresented OEF/OIF veterans’ drinking, and that other OEF/OIF veterans would think so as well. Participants felt they knew what their peers were consuming, and it was as much or more than their own consumption:

"[Veteran H]: *This one here is one of the ones that I thought, “Oh, bull.” “I drink more drinks in a single day than 99 percent of male VA patients my age.” I don’t believe that for a second, okay?… I mean, I know a lot of guys, a lot of Vets… you know, and, they drink just as much as I do, if not more.*"

### Veterans spontaneously offered unsolicited stories/anecdotes about drinking

Although the interview was designed to elicit participant opinions about DrinkCheck and not private information about themselves, every participant volunteered personal details about their experience with alcohol. Personal stories were interwoven throughout the interviews and were often the byproduct of a participant’s review of a specific e-SBI feature. In reference to “managing pain” listed among the health concerns, Veteran F shared this:

"*I’ll have to say, I do drink sometimes cause of that. Like, I separated my shoulder last week, and I can’t just go to the doctors anytime and pick up medication. And, so, a six pack helps. And, a six pack and a hot tub does a pretty good job on that one. So, I have to say I do drink for that sometimes.*"

Veterans also spoke openly about their experience with alcohol without responding to a specific e-SBI feature. In explaining his tolerance for alcohol, Veteran E offered:

"*My family has a history of alcoholism, and I remember being seven years old and having to drive my dad home drunk from the bar. At seven. It’s kind of screwy. Since then, my father has severely cut back on his drinking. He’s 74 now. He’ll go out, you know, maybe once, twice, a week.... He’ll go out with his buddy, and they’ll have six, seven beers… another night during the week, they’ll do the same thing.*"

### Veterans reported benefits of completing DrinkCheck

Despite elicited criticism regarding specific e-SBI features, all nine OEF/OIF veterans found the program somewhat helpful in encouraging consideration of their drinking. Specifically, some veterans appreciated being provided the recommended drinking limits [42] and felt the information succinctly summed up what they needed to know to avoid unsafe consumption: 

"[Veteran C]: *I’ve seen a whole bunch of, like, drinking videos and stuff like that, and they really don’t say, you know, what the actual limit is for, you know, not really harming yourself.*"

Further, despite some doubts and dissatisfaction with the feedback, a few OEF/OIF veterans expressed interest in changing their drinking:

"[Veteran A]: *You know, it definitely makes me reflect. I think, “Well, hold on a second, you know, it would be better not to drink quite so much at those times.”*"

Some were surprised by their consumption and suggested other veterans using the program might be as well:

"[Veteran G referring to normative feedback]: *I liked this page, because I was like “Oh my god” (laughs). Cause I just didn’t realize… putting it on the most that you drank in one night, I was like, “Wow, remind me not to do that very often.” I’ll think about that a lot more.*"

Lastly, several participants indicated interest in assistance with their drinking beyond what DrinkCheck had to offer:

"[Veteran D]: *I don’t want to stop drinking alcohol. And I think I can safely have a drink or two, but maybe I should look at some strategies. Not for quitting, ‘cause I saw* [the “Strategies for Cutting Down” link on the Resource page] *and I thought, “That’s it.”*"

In particular, Veteran B, who described a family history of problem drinking and experience with Alcoholics Anonymous, suggested offering additional resources:

"[Veteran B]: *Maybe resource links if the VA has resources… “These are healthy drinking habits. These are resources that are available to you to help you get there.”…[I]f it takes more of a, a corrective approach, giving them steps, giving them resources, giving them options… those types of things are going to cause people to want to come back, to want to re-evaluate themselves, to continue to use the system.*"

## Discussion

This qualitative observational study explored the acceptability of an efficacious web-based Australian e-SBI for college students [29] adapted for use with OEF/OIF veteran outpatients. Although each of the nine OEF/OIF veteran participants found different features of DrinkCheck useful, all felt it was helpful in promoting consideration of their own drinking. Findings that emerged from interviews included participant difficulty answering past-year consumption questions due to changes in drinking during deployment, doubts about the representativeness of the normative feedback from VA outpatients, a desire for more transparent personalized feedback on what made their reported drinking risky, and personalized risk-reduction strategies. During the interview, all participants spontaneously volunteered personal information about their drinking, and several expressed interest in changing their drinking and/or additional resources for reducing their consumption.

Some findings from this study are specific to development of e-SBIs for OEF/OIF veterans. Assessment of alcohol consumption should allow for possible recent changes in drinking. Some participants reported averaging their drinking over the entire year, including time when they were not drinking due to deployment. To avoid underestimation of recent alcohol use, future e-SBI adaptations for returned veterans may want to account for deployment periods by assessing recent drinking or using the AUDIT-C questions without a timeframe [43]. Additionally, OEF/OIF veterans may be more willing to reflect on their own drinking if normative feedback is based on comparable measures from other OEF/OIF veterans [44]. Normative feedback was based on the greatest number of drinks on one occasion from age- and gender-matched AUDIT-C data from VA outpatient surveys, while e-SBI participants were explicitly asked the greatest number of drinks in the past four weeks. This incongruence may have contributed to participant skepticism. Lastly, an anonymous e-SBI may be an effective tool for ensuring that OEF/OIF veterans receive feedback that is reflective of their actual drinking. Many participants felt they would be more likely to accurately report their alcohol use to an anonymous web-based program than in-person, consistent with previous reports from active-duty Marines [20]. Military personnel can lose their jobs for alcohol-related misconduct or failure to attend or respond to alcohol treatment [45], and a recent change to regulations [46] that allows for sharing of previously protected medical records between the VA and the US Department of Defense is likely to augment concerns about stigma and job-related impact [2,8].

Some findings from this study of nine OEF/OIF veterans may be applicable to the development of e-SBIs and possibly clinically delivered BIs for other populations. Results suggest that feedback needs to be transparent, as participants were nearly universal in their wish to know how they were assigned to their particular risk category. Tailored personal feedback could include a summary of a participant’s drinking and whether, and the extent to which, weekly or daily limits were exceeded. Electronically delivered SBIs could also be used to educate patients about the risks of heavy episodic drinking. Several participants felt their heavy drinking episodes were not problematic if they were not taking physical risks (e.g., driving) or experiencing adverse alcohol-related consequences. The e-SBI could inform such patients of the association of heavy episodic drinking with cognitive deficits [47] and development of addiction to alcohol [48,49].

Finally, this study suggests that following e-SBI with an opportunity for participants to debrief could serve as a method to engage veterans around their drinking. Although all nine OEF/OIF veterans easily engaged in a detailed candid discussion of the website as anticipated, it was not anticipated that participants would spontaneously volunteer personal information about their own drinking. This finding suggests that providing returning veterans anonymous opportunities to complete and then discuss their alcohol-use assessment and feedback may be a useful method for engaging them in conversations about their drinking.

Results of this study also suggest areas for further research. Normative feedback is a common component of e-SBI and, although no systematic review of the effectiveness of normative feedback has been conducted, it has reduced drinking in college students [44] and was a key component of THRIVE [29]. However, it remains unknown whether normative feedback has efficacy in general adult or clinical populations. Additionally, little is known about the usefulness of e-SBI for highest risk patients, including individuals with alcohol dependence [20,50]. Results from this study suggest that e-SBI should address the needs of these patients. Further, given the scarcity of qualitative research on patient experiences of clinically delivered BI, results also suggest the potential value of similar research on BI delivered clinically. Lastly, this research underscores the value of eliciting qualitative input on e-SBI from the target population.

This study has important limitations, the greatest being that enrollment was limited to a convenience sample of nine OEF/OIF veterans, all of whom were outpatients at a single medical center. Therefore, analyses were limited to descriptive exploration of responses, and it is unlikely that saturation of themes was reached. Further, the small sample was likely inadequate for observing the variation that exists among the general population of OEF/OIF veterans. It is possible that different findings may have emerged had purposive sampling been used to identify patients from different veteran subgroups, additional sites and regions, and veterans who were not receiving care from the VA. Results from this study may best be interpreted as a useful starting point for informing future development of e-SBI for OEF/OIF and other veterans.

## Conclusions

To summarize, this qualitative exploratory study of nine OEF/OIF veterans found that e-SBI was useful in promoting consideration of their own drinking. Such interventions may be most appealing to OEF/OIF veterans if they ensured anonymity, provided personalized transparent feedback about alcohol-related risk, consider the context for drinking, and provide strategies to reduce drinking and additional resources for veterans with more severe alcohol misuse. Results also highlight the importance of educating patients about the risks associated with heavy episodic drinking. Offering an e-SBI program that is relevant and attractive to OEF/OIF veterans could be an effective strategy for increasing their access to evidenced-based care for alcohol misuse.

## Appendix A DrinkCheck Patient Interview Prompts: General Topics

Topic 1: General Reactions to Web-based Screening and Intervention

 1.1 First to begin, can you tell me your general response and impression of the website?

 [Probe: How did you feel about receiving computerized feedback?]

 [Probe: How did you feel about the feedback you received?]

 [Probe: Did you ever feel uncomfortable, angry, or frustrated while working through the website?]

 [Probe: What did you think of your drinking compared to others? Surprised?]

 [Probe: Was the summary information about your drinking clear and credible enough?]

 [Probe: What information on the website was of interest or useful or new to you?]

 [Probe: What information on the website was uninteresting or bothersome?]

 [Probe: Did it cause you to think about your drinking?]

Topic 2: Barriers/Facilitators to Web-based Screening and Intervention

 1.1 What are your thoughts about a web-based intervention generally? For tobacco use? Alcohol use?

 [Probe: What would make you most likely want to use such a website?]

 [Probe: Do you have any concerns about answering questions about your alcohol use?]

 [Probe: Do you feel it would be easier to respond more openly to a computer than a VA provider?]

 [Probe: Is there anything about your drinking that you would feel comfortable sharing on the web that you would not be willing to share with your provider?]

 [Probe: Would you want to talk to your provider about the feedback you received from this website?]

 [Probe: Would you want your responses to this website made available to your provider?]

 1.2 Would you be willing to complete this web-based program in a kiosk in the waiting room of the clinic? Through MyHealtheVet?

[Probe: Would you be willing to complete the web-based program if you were not compensated for your time?]

Topic 3: Impressions of Web Page Layout

 2.1 What were your impressions of this page?

 [Probe: What did you like?]

 [Probe: What did you dislike?]

 [Probe: Which questions, if any, were hard to answer?]

 [Probe: Which feedback if any was unclear?]

 [Probe: Which questions or feedback bothered you]

 [Probe: What do you like or dislike about the tables/figures?]

 [Probe: Does the placement of tables/figures complement or distract from the text?]

 [Probe: What was difficult for you on this page, if anything?]

 [Probe: How do you think others would respond to this page?]

 [Probe: How could we improve this page?]

 [Probe: Is there anything we could add that would make it more credible, relevant or interesting to veterans like you?]

 [Probe: Is there anything about the pictures that you like or dislike?]

Topic 4: Reactions to Web Page Content

 3.1 What sort of reactions did you have when you read this page?

 [Probe: Is the content on this page useful, informative, or thought provoking?]

 [Probe: Is it relevant to you or other OEF/OIF veterans?]

 [Probe: Was the feedback based on peer drinking believable?]

 [Probe: Can you think of any other information it should include?]

Topic 5: General Questions

 4.1 In general, how was the website helpful to you?

 [Probe: How could you see it being helpful to others?]

 [Probe: Was there anything you’d like to see in the website that wasn’t there?]

 [Probe: What would help make it a better website?]

 [Probe: What did you think the program was going to be about?]

 [Probe: Did you want to stop the website? Where in the website did you want to stop?

 [Probe: What other health topics should the website include?]

 4.2 What thoughts do you have on how the VA should help veterans who drink an unhealthy amount of alcohol?

1) more web-based interventions

2) provide list of self-help or community resources

3) provide medications to reduce drinking

4) telephone counseling

5) review of recovery goals and commitment to change

6) individual counseling

## Competing interests

We certify that none of the authors has an affiliation with, or financial involvement in, any organization or entity with a direct financial interest in the subject matter or materials discussed in the manuscript (e.g., employment, consultancies, stock, ownership, honoraria). We declare that no financial or nonfinancial competing interests exist concerning the authors and the contents of this article.

## Authors’ contributions

All authors made substantial contributions to the execution of the study, have been involved from the beginning in drafting the manuscript, and have given approval of the final draft. Specifically, GTL carried out project management, completed patient interviews, participated in iterative review of patient interview coding, and drafted the manuscript. EJH participated in study design, coded the patient interviews, participated in iterative review of patient interview coding, and helped draft the manuscript. LJC coded patient interviews, participated in iterative review of patient interview coding, and helped draft the manuscript. CEL participated in study design, iterative review of patient interview coding, and helped draft the manuscript. ECW participated in study design, iterative review of patient interview coding, and helped draft the manuscript. RMT reviewed transcripts and coding for accuracy, participated in iterative review of patient interview coding, and helped draft the manuscript. EJL provided expertise on qualitative study design and analyses, participated in iterative review of patient interview coding, and helped draft the manuscript. KK provided the web-interface and programming of the Australian e-SBI for adaptation in this study as well as expertise on study design and e-SBI development, participated in iterative review of patient interview coding, and helped draft the manuscript. SCH provided access to the Deployment Health Clinic for patient recruitment as well as expertise on OEF/OIF veterans, participated in study design and review of patient interview coding, and helped draft the manuscript. KAB conceived of the study, obtained funding, and oversaw and participated in all aspects of the study including drafting of the manuscript. All authors read and approved the final manuscript.

## References

[B1] Defense Manpower Data Center CTS Deployment File Baseline Report for Operation Enduring Freedom and Operation Iraqi Freedom 2011 US Department of Defense, Washington, DC

[B2] Hoge CW Castro CA Messer SC McGurk D Cotting DI Koffman RL Combat duty in Iraq and Afghanistan, mental health problems, and barriers to care N Engl J Med 2004 351 13 22 10.1056/NEJMoa040603 15229303

[B3] Jacobson IG Ryan MA Hooper TI Smith TC Amoroso PJ Boyko EJ Gackstetter GD Wells TS Bell NS Alcohol use and alcohol-related problems before and after military combat deployment JAMA 2008 300 663 675 10.1001/jama.300.6.663 18698065 PMC2680184

[B4] Milliken CS Auchterlonie JL Hoge CW Longitudinal assessment of mental health problems among active and reserve component soldiers returning from the Iraq war JAMA 2007 298 2141 2148 10.1001/jama.298.18.2141 18000197

[B5] Calhoun PS Elter JR Jones ER Kudler H Straits-Troster K Hazardous alcohol use and receipt of risk-reduction counseling among U.S. veterans of the wars in Iraq and Afghanistan J Clin Psychiatry 2008 69 1686 1693 10.4088/JCP.v69n1103 19012816

[B6] Hawkins EJ Lapham GT Kivlahan DR Bradley KA Recognition and management of alcohol misuse in OEF/OIF and other veterans in the VA: a cross-sectional study Drug Alcohol Depend 2010 109 147 153 10.1016/j.drugalcdep.2009.12.025 20167440

[B7] McDevitt-Murphy ME Williams JL Bracken KL Fields JA Monahan CJ Murphy JG PTSD symptoms, hazardous drinking, and health functioning among U.S.OEF and OIF veterans presenting to primary care J Trauma Stress 2010 23 108 111 20104586 10.1002/jts.20482 PMC2876344

[B8] Pietrzak RH Johnson DC Goldstein MB Malley JC Southwick SM Perceived stigma and barriers to mental health care utilization among OEF-OIF veterans Psychiatr Serv 2009 60 1118 1122 10.1176/appi.ps.60.8.1118 19648201

[B9] Erbes CR Curry KT Leskela J Treatment Presentation and Adherence of Iraq/Afghanistan Era Veterans in Outpatient Care for Posttraumatic Stress Disorder Psychol Serv 2009 6 175 183

[B10] Kaner E Beyer F Dickinson H Pienaar E Campbell F Schlesinger C Heather N Saunders J Burnand B Effectiveness of brief alcohol interventions in primary care populations Cochrane Database Syst Rev 2007 CD004148 10.1002/14651858.CD004148.pub3 17443541

[B11] Bradley KA Williams EC Achtmeyer CE Volpp B Collins BJ Kivlahan DR Implementation of evidence-based alcohol screening in the Veterans Health Administration Am J Manag Care 2006 12 597 606 17026414

[B12] Lapham GT Achtmeyer CE Williams EC Hawkins EJ Kivlahan DR Bradley KA Increased documented brief alcohol interventions with a performance measure and electronic decision support Med Care 2012 50 179 187 10.1097/MLR.0b013e3181e35743 20881876

[B13] Bradley KA Lapham GT Hawkins EJ Achtmeyer CE Williams EC Thomas RM Kivlahan DR Quality concerns with routine alcohol screening in VA clinical settings J Gen Intern Med 2011 26 299 306 10.1007/s11606-010-1509-4 20859699 PMC3043188

[B14] Ouimette PC Vogt D Wade M Tirone V Greenbaum MA Kimerling R Laffaye C Fitt JE Rosen CS Perceived barriers to care among veterans health administration patients with posttraumatic stress disorder Psychol Serv 2011 8 212 223

[B15] Linke S Murray E Butler C Wallace P Internet-based interactive health intervention for the promotion of sensible drinking: patterns of use and potential impact on members of the general public J Med Internet Res 2007 9 e10 10.2196/jmir.9.2.e10 17513281 PMC1874715

[B16] Cunningham JA Humphreys K Koski-Jannes A Providing personalized assessment feedback for problem drinking on the Internet: a pilot project J Stud Alcohol 2000 61 794 798 11188484 10.15288/jsa.2000.61.794

[B17] Saitz R Helmuth ED Aromaa SE Guard A Belanger M Rosenbloom DL Web-based screening and brief intervention for the spectrum of alcohol problems Prev Med 2004 39 969 975 10.1016/j.ypmed.2004.04.011 15475031

[B18] Gerbert B Bronstone A Pantilat S McPhee S Allerton M Moe J When asked, patients tell: disclosure of sensitive health-risk behaviors Med Care 1999 37 104 111 10.1097/00005650-199901000-00014 10413398

[B19] Straits-Troster K Kudler H Jones E Calhoun P Executive Summary Report on Focus Groups: Clinical Needs Assessment of Operation Iraqi Freedom (OIF) and Operation Enduring Freedom (OEF) Veterans and Families 2007 VISN 6 Mental Illness Research, Education and Clinical Center (MIRECC), Durham, NC

[B20] Simon-Arndt CM Hurtado SL Patriarca-Troyk LA Acceptance of Web-based personalized feedback: user ratings of an alcohol misuse prevention program targeting U.S. Marines Health Commun 2006 20 13 22 10.1207/s15327027hc2001_2 16813485

[B21] Curry SJ eHealth research and healthcare delivery beyond intervention effectiveness Am J Prev Med 2007 32 S127 130 10.1016/j.amepre.2007.01.026 17466817

[B22] Bewick BM Trusler K Mulhern B Barkham M Hill AJ The feasibility and effectiveness of a web-based personalised feedback and social norms alcohol intervention in UK university students: a randomised control trial Addict Behav 2008 33 1192 1198 10.1016/j.addbeh.2008.05.002 18554819

[B23] Copeland J Martin G Web-based interventions for substance use disorders: a qualitative review J Subst Abuse Treat 2004 26 109 116 10.1016/S0740-5472(03)00165-X 15050088

[B24] Elliott JC Carey KB Bolles JR Computer-based interventions for college drinking: a qualitative review Addict Behav 2008 33 994 1005 10.1016/j.addbeh.2008.03.006 18538484 PMC2441945

[B25] Carey KB Henson JM Carey MP Maisto SA Computer versus in-person intervention for students violating campus alcohol policy J Consult Clin Psychol 2009 77 74 87 19170455 10.1037/a0014281 PMC2657221

[B26] Rooke S Thorsteinsson E Karpin A Copeland J Allsop D Computer-delivered interventions for alcohol and tobacco use: a meta-analysis Addiction 2010 105 1381 1390 10.1111/j.1360-0443.2010.02975.x 20528806

[B27] Khadjesari Z Murray E Hewitt C Hartley S Godfrey C Can stand-alone computer-based interventions reduce alcohol consumption? A systematic review Addiction 2011 106 267 282 10.1111/j.1360-0443.2010.03214.x 21083832

[B28] Kypri K Langley JD Saunders JB Cashell-Smith ML Herbison P Randomized controlled trial of web-based alcohol screening and brief intervention in primary care Arch Intern Med 2008 168 530 536 10.1001/archinternmed.2007.109 18332300

[B29] Kypri K Hallett J Howat P McManus A Maycock B Bowe S Horton NJ Randomized controlled trial of proactive web-based alcohol screening and brief intervention for university students Arch Intern Med 2009 169 1508 1514 10.1001/archinternmed.2009.249 19752409

[B30] Babor TF de la Fuente JR Saunders J Grant M AUDIT. Alcohol Use Disorders Identification Test: guidelines for use in primary health care 1989 World Health Organization, Geneva

[B31] Kypri K Saunders JB Williams SM McGee RO Langley JD Cashell-Smith ML Gallagher SJ Web-based screening and brief intervention for hazardous drinking: a double-blind randomized controlled trial Addiction 2004 99 1410 1417 10.1111/j.1360-0443.2004.00847.x 15500594

[B32] Miller WR Rollnick S Motivational Interviewing: Preparing People to Change Addictive Behavior 1991 Guilford Press, New York

[B33] Hallett J Maycock B Kypri K Howat P McManus A Development of a Web-based alcohol intervention for university students: processes and challenges Drug Alcohol Rev 2009 28 31 39 10.1111/j.1465-3362.2008.00008.x 19320673

[B34] Bradley KA Williams EC Achtmeyer CE Hawkins EJ Harris AH Frey MS Craig T Kivlahan DR Measuring performance of brief alcohol counseling in medical settings:a review of the options and lessons from the Veterans Affairs (VA) health care system Subst Abus 2007 28 133 149 10.1300/J465v28n04_05 18077309

[B35] Wright SM Craig T Campbell S Schaefer J Humble C Patient satisfaction of female and male users of Veterans Health Administration services J Gen Intern Med 2006 21 Suppl 3 S26 32 16637941 10.1111/j.1525-1497.2006.00371.x PMC1513173

[B36] Borson S Scanlan J Brush M Vitaliano P Dokmak A The mini-cog: a cognitive 'vital signs' measure for dementia screening in multi-lingual elderly Int J Geriatr Psychiatry 2000 15 1021 1027 10.1002/1099-1166(200011)15:11<1021::AID-GPS234>3.0.CO;2-6 11113982

[B37] US Chief Information Officers Council: Paperwork Reduction Act of 1995 1995 [http://www.cio.gov/documents/paperwork_reduction_act_1995.html]

[B38] US Department of Veterans Affairs: VA Handbook 6310.3 [http://www.va.gov/vapubs/viewPublication.asp?Pub_ID=36&FType=2]

[B39] Gask L Ludman E Schaefer J Qualitative study of an intervention for depression among patients with diabetes: how can we optimize patient-professional interaction? Chronic Illn 2006 2 231 242 17007699 10.1177/17423953060020030401

[B40] King N Symon G, Cassell C Template analysis Qualitative Methods and Analysis in Organizational Research: A Practical Guide 1998 Sage, Thousand Oaks, CA 118 134

[B41] Saitz R Palfai TP Freedner N Winter MR Macdonald A Lu J Ozonoff A Rosenbloom DL Dejong W Screening and brief intervention online for college students: the ihealth study Alcohol Alcohol 2007 42 28 36 17130139 10.1093/alcalc/agl092

[B42] US National Institute on Alcohol Abuse and Alcoholism: Helping Patients Who Drink Too Much: A Clinician’s Guide (Updated 2005 Edition) [http://pubs.niaaa.nih.gov/publications/Practitioner/CliniciansGuide2005/clinicians_guide.htm]

[B43] Bradley KA DeBenedetti AF Volk RJ Williams EC Frank D Kivlahan DR AUDIT-C as a brief screen for alcohol misuse in primary care Alcohol Clin Exp Res 2007 31 1208 1217 10.1111/j.1530-0277.2007.00403.x 17451397

[B44] Lewis MA Neighbors C Optimizing personalized normative feedback: the use of gender-specific referents J Stud Alcohol Drugs 2007 68 228 237 17286341 10.15288/jsad.2007.68.228 PMC2459320

[B45] Management of Substance Use Disorders Working Group: VA/DoD Clinical Practice Guideline for Management of Substance Use Disorders[http://guideline.gov/content.aspx?id=15676]

[B46] US Federal Register: Interim Final Rule. Sharing Information Between the Department of Veterans Affairs and the Department of Defense (38 CFR Part 1) [http://www.va.gov/ORPM/docs/20111020_AN95_SharingInformationBetweentheDVAandDOD.pdf] 22013621

[B47] Maurage P Joassin F Speth A Modave J Philippot P Campanella S Cerebral effects of binge drinking: respective influences of global alcohol intake and consumption pattern Clin Neurophysiol 2012 123 892 901 10.1016/j.clinph.2011.09.018 22055841

[B48] Dawson DA Grant BF Li TK Quantifying the risks associated with exceeding recommended drinking limits Alcohol Clin Exp Res 2005 29 902 908 10.1097/01.ALC.0000164544.45746.A7 15897737

[B49] Saha TD Stinson FS Grant BF The role of alcohol consumption in future classifications of alcohol use disorders Drug Alcohol Depend 2007 89 82 92 10.1016/j.drugalcdep.2006.12.003 17240085 PMC2727876

[B50] Cunningham JA Humphreys K Kypri K van Mierlo T Formative evaluation and three-month follow-up of an online personalized assessment feedback intervention for problem drinkers J Med Internet Res 2006 8 e5 10.2196/jmir.8.2.e5 16867968 PMC1550700

